# Halicin: A New Horizon in Antibacterial Therapy against Veterinary Pathogens

**DOI:** 10.3390/antibiotics13060492

**Published:** 2024-05-27

**Authors:** Shuge Wang, Ke Zhao, Ziqi Chen, Dejun Liu, Shusheng Tang, Chengtao Sun, Hongliang Chen, Yang Wang, Congming Wu

**Affiliations:** 1 National Key Laboratory of Veterinary Public Health and Safety, College of Veterinary Medicine, China Agricultural University, Beijing 100094, China; b20203050362@cau.edu.cn (S.W.); s20223050931@cau.edu.cn (K.Z.); s20213050789@cau.edu.cn (Z.C.); liudejun@cau.edu.cn (D.L.); tssfj@cau.edu.cn (S.T.); sct@cau.edu.cn (C.S.); 2 School of Life Sciences, Xiamen University, Xiamen 361005, China; chenghl@vangenes.com; 3 Xiamen Vangenes Biotechnology Co., Ltd., Xiamen 361006, China

**Keywords:** halicin, pathogenic bacteria, veterinary medicine, PAE, PA-SME, toxicity, in vitro and in vivo antibacterial efficacy

## Abstract

It is crucial to discover novel antimicrobial drugs to combat resistance. This study investigated the antibacterial properties of halicin (SU3327), an AI-identified anti-diabetic drug, against 13 kinds of common clinical pathogens of animal origin, including multidrug-resistant strains. Employing minimum inhibitory concentration (MIC) and minimum bactericidal concentration (MBC) assessments, halicin demonstrated a broad-spectrum antibacterial effect. Time-killing assays revealed its concentration-dependent bactericidal activity against *Escherichia coli* ATCC 25922 (*E. coli* ATCC 25922), *Staphylococcus aureus* ATCC 29213 (*S. aureus* ATCC 29213), and *Actinobacillus pleuropneumoniae* S6 (*APP* S6) after 4 h of treatment at concentrations above the MIC. Halicin exhibited longer post-antibiotic effects (PAEs) and sub-MIC effects (PA-SMEs) for *E. coli* 25922, *S. aureus* 29213, and *APP* S6 compared to ceftiofur and ciprofloxacin, the commonly used veterinary antimicrobial agents, indicating sustained antibacterial action. Additionally, the results of consecutive passaging experiments over 40 d at sub-inhibitory concentrations showed that bacteria exhibited difficulty in developing resistance to halicin. Toxicology studies confirmed that halicin exhibited low acute toxicity, being non-mutagenic, non-reproductive-toxic, and non-genotoxic. Blood biochemical results suggested that halicin has no significant impact on hematological parameters, liver function, and kidney function. Furthermore, halicin effectively treated respiratory *A. pleuropneumoniae* infections in murine models. These results underscore the potential of halicin as a new antibacterial agent with applications against clinically relevant pathogens in veterinary medicine.

## 1. Introduction 

The extensive use of existing antimicrobial drugs, along with recent challenges in developing novel antimicrobial agents, has exacerbated the occurrence of antimicrobial resistance (AMR) among bacteria [1,2]. The misuse of antimicrobial agents has led to a worsening crisis of bacterial resistance, particularly in recent years, with veterinary clinics increasingly encountering antimicrobial-resistant bacterial infections and few effective treatments available [3]. Additionally, the use of low-dose antimicrobials as growth promoters in animal feed has intensified AMR issues globally. More alarmingly, AMR developed in animals can transfer to humans through the food chain and the environment, posing serious threats to public health safety [4,5].

The resistance profiles of porcine *Actinobacillus pleuropneumoniae* (*A. pleuropneumoniae*) strains have significantly increased in recent years. Archambault et al. [6] identified 43 *A. pleuropneumoniae* strains isolated from Canadian pigs, demonstrating high resistance to chlortetracycline (88.4%) and oxytetracycline (90.7%); 5 strains exhibited multidrug resistance to streptomycin, sulfonamide, penicillin, and tetracycline antibiotics, while 3 strains showed multidrug resistance to sulfonamide, streptomycin, and tetracycline antibiotics. Pereira et al. [7] determined the antimicrobial activity of 21 strains of *A. pleuropneumoniae* isolated from diseased pigs and found that 95% showed multidrug resistance to aminoglycosides, 81% to aminophenols, 76% to sulfonamides, 33% to β-lactams, and 14% to quinolones. Furthermore, Burch and Sperling in 2018 [8] noted a reduced susceptibility to amoxicillin in 11.3% of *A. pleuropneumoniae* strains. Extensive use of florfenicol has led to resistance levels among *A. pleuropneumoniae* isolates reaching as high as 34% [9]. This situation underscores the urgent need for the development of more efficient drugs.

Advances in computational approaches, notably artificial intelligence (AI), have not only opened new avenues for repositioning existing drugs for antimicrobial purposes [10], but also shown promise in identifying potential new antimicrobial agents [11], offering a strategic pathway to combat resistant bacteria. The use of AI to identify novel antimicrobial compounds through drug databases has emerged as a trend and has shown notable progress [12,13]. Through machine learning analysis of the “Drug Repurposing Hub”, a collection of nearly 1.07 billion chemical compounds, researchers identified “halicin” (originally SU3327), an anti-diabetic drug and a c-Jun N-terminal protein kinase (JNK) inhibitor, as possessing significant antibacterial activity [14]. Further studies have shown that halicin exhibited potent growth-inhibitory activity against *Escherichia coli* and possessed antibacterial effects against *Staphylococcus aureus*, *Mycobacterium tuberculosis,* and carbapenem-resistant *Enterobacteriaceae*. Additionally, halicin has demonstrated effective therapeutic action against mouse intestinal infections caused by *Clostridioides difficile* and skin infections caused by pan-resistant *Acinetobacter baumannii*. Notably, the WHO has listed *A. baumannii* as one of the top pathogens urgently requiring new antimicrobial treatments [15,16]. Furthermore, halicin has been demonstrated to have strong anti-biofilm activity against *S. aureus* [17]. It has been reported that halicin disrupted the bacterial membrane electrochemical gradient and upregulated bacterial genes associated with iron homeostasis, consequently inducing perturbations in △pH regulation across the bacterial cell membrane and ultimately arresting bacterial growth [14,18,19]. Consequently, the bacteria might be incapable of developing resistance to this novel mode of action [20]. 

Current research on the antibacterial activity of halicin has been limited to a few human-derived bacterial strains and lacks comprehensive antibacterial studies on pathogens relevant to veterinary clinical settings. Building upon recent findings, we hypothesized that halicin had potential for application in veterinary clinics. However, the antimicrobial activity and spectrum of halicin against common animal pathogens have not been investigated, nor have its specific antimicrobial properties and in vivo safety been elucidated. Systematic research is required to develop halicin as a novel antimicrobial agent for the treatment of veterinary pathogens. This study aimed to bridge this gap by evaluating the antibacterial performance of halicin both in vitro and in vivo, and by assessing its in vivo toxicity, thus providing a comprehensive understanding of its potential as an antibacterial agent.

## 2. Materials and Methods

### 2.1. Materials

Halicin was obtained from Hubei Norna Technology Co., Ltd. (Wuhan, Hubei, China), and ceftiofur and ciprofloxacin standards were purchased from Dr. Ehrenstorfer (Augsburg, Germany). Gentamicin, tetracycline, florfenicol, and trimethoprim/sulfamethoxazole were purchased from the China Institute of Veterinary Drug Control (Beijing, China). All chemical reagents and organic solvents were of analytical reagents (AR) or HPLC grade. All strains were cultured in relevant media such as Brain Heart Infusion Broth (BHI), Luria Broth (LB), Mueller–Hinton Broth (MHB), Tryptic Soy Broth (TSB), and modified *mycoplasma* medium base (Frey). All culture media were purchased from Solabio Biotech Co., Ltd. (Beijing, China). Distilled water generated by Milli Q (Millipore Corporation, Bedford, MA, USA) was used throughout this study.

The isolated strains used for the sensitivity assessment of halicin in this study consisted of 13 types encompassing both Gram-positive and -negative bacteria, as well as anaerobic and aerobic strains, including *E. coli* (isolated from pig and chicken), *Salmonella typhimurium* (pig and cattle), *Klebsiella pneumoniae* (cattle), *Staphylococcus* spp. and *Streptococcus* spp. (bovine mastitis), *Streptococcus suis* (pig), *Pasteurella multocida* (pig and cattle), *A. pleuropneumoniae* (pig), *Clostridium perfringens* (pig and chicken), *Haemophilus parasuis* (pig), *Pseudomonas aeruginosa* (quails), *A. baumannii* (pigs), and *Mycoplasma* (chickens) (20 of each pathogen). These strains were derived from typical food animals, including pig, chicken, and cattle, and were sourced from diverse farms, ensuring a representative sample. The model strains, including *E. coli* ATCC 25922, *S. aureus* ATCC 29213, and *S. typhimurium* strains TA_97_, TA_98_, TA_100_, and TA_102_, along with all isolated strains, were stored at the College of Veterinary Medicine of China Agricultural University. The strains, except for *Mycoplasma*, were identified using matrix-assisted laser desorption/ionization time-of-flight mass spectrometry (MALDI-TOF-MS; Antu Biotechnology Co., Ltd., Zhengzhou, Henan, China). Polymerase Chain Reaction (PCR) was used to identify *Mycoplasma* with MG-specific primers MG14F (5’-GCTTCCTTGCGGTTAGCAAC-3’) and MG13R (5’-GAGCTAATCTGTAAAGTTGGTC-3’), producing an amplified fragment of 185 bp [21]. The cycling protocol included an initial denaturation at 94 °C for 5 min, followed by 34 cycles of 94 °C for 45 s, annealing at 55 °C for 45 s, primer extension at 72 °C for 60 s, and a final extension at 72 °C for 5 min. The amplified products were analyzed using 1.5% agarose gel electrophoresis.

### 2.2. Antimicrobial Susceptibility Test

The MIC of the strains, except for *Mycoplasma*, was determined following the CLSI-recommended microdilution method [22]. The antimicrobials used in this study were halicin, ceftiofur, gentamicin, tetracycline, ciprofloxacin, florfenicol, and trimethoprim/sulfamethoxazole. The drugs were serially diluted (128–0.25 μg/mL) in MHB and added to 96-well plates with bacteria (1 × 10^6^ CFU/mL). Positive (bacteria present, no drug) and negative controls (medium only) were included. Following an 18–22 h incubation at 37 °C, the MIC, defined as the minimum concentration without visible growth, was determined based on solution turbidity. Additionally, the minimum bactericidal concentration (MBC) was established as the lowest concentration with no visible bacterial colonies after 24 h at 37 °C on agar plates.

The in vitro susceptibility of *Mycoplasma* to antimicrobials was assessed by the color change unit (CCU) method [23]. The drugs were serially diluted (128–0.25 μg/mL) in modified FM-4 medium and added to 96-well plates. Serial tenfold dilutions of the strains were prepared in modified FM-4 liquid medium, following previously reported formulas [21], to a titer of 10^5^ CCU/mL and then added to the bacterial culture plates. Positive and negative controls were included. The plate was sealed with sterile medical tape and incubated at 37 °C with 5% CO_2_ for 5 d. The MIC was recorded as the highest dilution without a color change, indicated by yellow positive control wells and unchanged negative control wells (orange). The MBC was determined as the lowest concentration that inhibited bacterial growth for 168 h at 37 °C with 5% CO_2_. The measurement was repeated three times.

### 2.3. The Time-Kill Assays

*E. coli* ATCC 25922, *S. aureus* ATCC 29213, and *APP* S6 were incubated in BHI medium (the cultivation of *APP* S6 requires the addition of 5% fetal bovine serum and 1% NAD) overnight and grown to the log phase at 37 °C, 200 rpm. The bacterial suspensions were diluted to 1.5 × 10^6^ CFU/mL with fresh broth. The in vitro time-killing curves of halicin in MHB medium were determined by monitoring the changes in colony counts from 100 μL samples on agar plates during incubation under a series of halicin concentrations (1/2 to 8 × MIC) over a continuous time period (0, 1, 2, 4, 6, 8, 10, 12 and 24 h), while 0.1 M PBS served as the control. The killing curves were determined in triplicate for each concentration.

### 2.4. Determination of the Post-Antibiotic Effect (PAE) and Post-Antibiotic Sub-MIC Effect (PA-SME)

The PAE was determined following established methods [24]. The antibacterial drug at ten times the MIC was added to 10 mL MHB tubes containing organisms in logarithmic growth (10^10^ CFU/mL). Two control tubes were included: one with antibiotic-free MHB and the second, to ensure effective antibiotic removal, containing the drug at a 1:1000 dilution. Incubation occurred at 37 °C for 2 h. Post incubation, the antibiotic was removed by diluting the drugs at a 1:1000 ratio with MHB. Colony counts were assessed before (T2) and after 2 h of antibiotic exposure (T1), immediately after washing (time 0), and every 2 h until visible turbidity occurred. Microorganisms were diluted in 0.1 M PBS, and 100 μL was plated onto agar plates, with colonies counted after 24 h in a 37 °C incubator.

The PAE is defined as PAE = T − C, where T is the time required for the CFU count in the test culture to increase by 1 log_10_ above the count observed at time 0, and C represents the corresponding time for the antibiotic-free control.

To determine the PA-SME [25], the PAE was performed as described above with 10 × MIC of halicin, ceftiofur, or ciprofloxacin. After a 2 h incubation, the drug was removed, and the sample was divided into 4 tubes, 3 of which contained the antibiotic at 0.1, 0.2, and 0.3 times the MIC. The 4th tube served as the control and contained only MHB without the drug. The remaining steps are consistent as mentioned above.

The PA-SME is defined as PA-SME = Tpa − C, where Tpa is the time required for the cultures, previously exposed to antibiotics and then re-exposed to various sub-MIC concentrations, to increase by 1 log_10_ above the count obtained immediately after antibiotic removal. C represents the corresponding time for the unexposed control. The experiment was conducted three times.

### 2.5. The Minimum Preventable Concentration (MPC) and the Mutant Selection Window (MSW) Determination

The MPC was determined according to the standard agar dilution method [26,27,28]. A bacterial suspension (10^10^ CFU/mL) was plated (100 μL) on agar plates with various drug concentrations (0, 1, 2, 4, 8, 16, and 64 × MIC) and incubated at 37 °C for 24 h. The initial provisional mutant prevention concentration (MPCpr) was defined as the lowest drug concentration that inhibited colony growth after 24 h. Subsequently, drug concentrations were decreased by 20% from MPCpr to prepare agar plates for further MPC determination. The process was repeated, and the final MPC was identified as the lowest drug concentration with no colony growth after 24 h of incubation. The range of concentrations between the MIC and the MPC is defined as the MSW.

### 2.6. Induction of Halicin-Resistant Mutant Strains

For serial passage evolution, a single colony of *E. coli* ATCC 25922, *S. aureus* ATCC 29213, or *APP* S6 was cultured in 1 mL of medium for 4–6 h to reach the logarithmic growth phase. The suspensions were diluted 1:1000 into 10 mL of fresh MHB with antibacterial drugs at concentrations of 0.25, 0.5, 1, 2, and 4 × MIC. After incubating at 37 °C for 24 h, MIC values were recorded. Bacteria thriving in the highest concentration of halicin (ciprofloxacin or ceftiofur) were diluted into fresh MHB at 1:1000 from a dense culture and reintroduced to halicin (ciprofloxacin or ceftiofur) at two-fold serial dilutions. This cycle was repeated every 24 h for 40 d, with daily MIC detection to monitor potential drug resistance.

### 2.7. Acute Toxicity Test of Mice Treated with Halicin

A total of 330 specific pathogen-free (SPF) ICR mice (25–30 g) were obtained from Sibeifu Biotechnology Co., Ltd. (Beijing, China). All animal care and testing procedures adhered to the Beijing public service facilities’ guidelines for laboratory animal care and utilization, under approved registration number Aw81213202-2-6.

Acute intraperitoneal and oral toxicity test: 120 SPF ICR mice were randomly divided into 12 groups (10 mice per group, half males and half females). Intraperitoneal injections were administered to six groups of mice at doses of 80.00, 60.65, 45.98, 34.8, 26.43, and 20.40 mg/kg b.w., respectively. For the acute oral toxicity test, the test substance was administered at doses of 512.64, 808.43, 1274.90, 2010.51, 3170.58, and 5000.00 mg/kg b.w. to another six groups of mice, each receiving 0.2 mL/10 g b.w. of the test substance via stomach tubes, respectively. Mice were monitored for behavior, toxicity signs, and mortality (criteria in Table S1) until the 28th day of experimentation. Necropsies were conducted on deceased mice. If no mortality was observed, two animals were randomly selected from each group for gross examination at necropsy at the end of this research.

Ames test: The regulatory Ames test was conducted in compliance with OECD 471 [29]. The experiment involved 5 dose groups (0.261, 0.0522, 0.01044, 0.002088, 0.0004176 μg/dish), two negative controls (sterile water, DMSO), and one positive control (2-aminofluorene, fenaminosulf, or sodium azide). TA_97_, TA_98_, TA_100_, and TA_102_ were divided into eight treatment groups, with or without the S9 metabolic activation system. As described in the literature [16,30], solutions of the test substance (or negative/positive control) and bacterial suspension (with/without 0.5 mL of 10% S9) were successively added into the plate containing 2 mL of agar and then incubated at 37 °C for 48 h. The experiments were performed in triplicate.

Sperm abnormality test: 50 male SPF ICR mice weighing 25–35 g were randomly divided into 5 groups, with 10 mice in each group. The treatment groups received oral doses of 159.36, 79.68, and 39.84 mg/kg b.w. for 5 consecutive days, respectively. Mice in the positive and negative controls received 40 mg/kg b.w. of cyclophosphamide and 40 mg/kg b.w. of 0.5% carboxymethyl cellulose sodium, respectively, each delivered as 0.2 mL/10 g b.w. After 35 d, the epididymides were collected, and sperm morphology was examined in each dose group, with 1000 sperm analyzed for abnormalities [31].

Bone marrow micronucleus test: The in vivo bone marrow micronucleus test in mice was conducted according to a previously described standardized protocol [32]. A total of 80 SPF ICR mice weighing 25–30 g were divided into 5 groups of 16 (equally distributed by gender). Experimental groups received doses of 637.45, 318.73, and 159.36 mg/kg b.w., each administered as 0.2 mL/10 g b.w. The positive control (cyclophosphamide for 40 mg/kg b.w.) and negative control (0.5% carboxymethyl cellulose sodium) were included. Mice were orally administered 0.2 mL/10 g b.w. once a day for 2 d. Subsequently, 6 mice (equal numbers of males and females) were randomly selected from each group for euthanasia, followed by bone marrow sampling and then slide preparation stained with Giemsa. Micronuclei were counted in over 1000 cells, including mature red blood cells (RBCs), to record polychromatic erythrocyte (PCE) and micro-nucleated PCE cell counts, as well as total RBC counts per mouse.

Chromosomal aberration test on bone marrow cells: This test was performed according to OECD GLP guideline 473 [33], and the methods were described previously with some minor modifications [34]. Briefly, 80 SPF ICR mice weighing 25–30 g were randomly divided into 5 groups of 16 (equally distributed by gender), and they received halicin at doses of 637.45 mg/kg b.w., 318.73 mg/kg b.w., and 159.36 mg/kg b.w., cyclophosphamide (40 mg/kg b.w., positive group), and 0.5% carboxymethyl cellulose sodium (negative control) once daily for 3 d via oral gavage, each administered as 0.2 mL/10 g b.w., respectively. A total of 6 mice (equally distributed by gender) were euthanized 24 h after the last dose via cervical dislocation, and femoral bone marrow cells were prepared for slide examination. Chromosomal abnormalities and numerical anomalies were observed and recorded under a double-blind method.

### 2.8. In Vivo Toxicity Assessment

A total of 40 SPF female BALB/C mice weighing 18–22 g (N = 5 per group) received halicin orally at doses of 25.48, 12.74, and 6.37 mg/kg b.w., and via intraperitoneal (i.p.) injections at doses of 3.69, 1.47, and 0.74 mg/kg b.w. The PBS (0.1 M) groups were also included. After 24 h, blood samples were collected from the ophthalmic venous plexus for hematological analysis, including white blood cell (WBC), red blood cell (RBC), hemoglobin (HGB), and platelet (PLT) counts. Serum obtained by centrifugation at 4000 rpm for 10 min was analyzed for alanine transaminase (ALT), aspartate transaminase (AST), and blood urea nitrogen (BUN) concentrations using an Auto-Analyzer (Architect C-800, Abbott Diagnostic Systems, Abbott Park, IL, USA). The mice were then euthanized, and organs (heart, liver, spleen, lung, and kidney) were excised, fixed with 15 mL of 4% neutral paraformaldehyde fixative solution, stained with H&E, and imaged under a microscope.

### 2.9. Mouse Pneumonia Infection Model

A total of 48 SPF female BALB/C mice were randomly divided into 8 groups: infection group (*APP* S6), control group (0.1 M PBS), and administration groups (H1, M1, and L1 at doses of 25.48 mg/kg b.w., 12.74 mg/kg b.w., and 6.37 mg/kg b.w., respectively), as well as intraperitoneal injection groups (H2, M2, and L2 at doses of 3.69 mg/kg b.w., 1.47 mg/kg b.w., and 0.74 mg/kg b.w., respectively). The pneumonia model was induced by the intranasal administration of 1.2 × 10^8^ CFU/mL of *APP* S6 suspension. Clinical signs, including appetite, respiratory rate, mental state, and nasal secretions, were monitored and scored based on Table S2 [35]. At 48 h post administration, the surviving mice were euthanized by cervical dislocation. Serial dilutions of various sample suspensions were plated on TSA agar. Additionally, another 48 mice that received the same treatment were used for clinical symptom scoring observations.

### 2.10. Statistical Analysis

Data analysis was performed using GraphPad Prism 9.0 (Software Inc., San Diego, CA, USA). Additionally, a two-independent-samples *t*-test was applied to compare two groups, and one-way ANOVA followed by post hoc Bonferroni’s correction were performed for all the variables among multiple groups. Results with *p* < 0.05 were considered statistically significant.

## 3. Results and Discussion

### 3.1. Halicin Exhibited Potent Antibacterial Activity against Animal Pathogens

AMR poses a global health crisis, stemming from the rapid increase in multidrug-resistant bacteria and the prolonged development of new antimicrobials [36,37,38]. In response, AI, specifically machine learning, is considered a potential solution to impede the spread of AMR [39,40,41]. AI offers effective strategies for predicting and identifying AMR in bacteria. Furthermore, the synergy between machine learning algorithms and laboratory testing can accelerate the discovery of novel antimicrobials [42,43]. The application of machine learning led to the discovery of halicin as the first innovative broad-spectrum antimicrobial, showing structural similarity to metronidazole (Tanimoto similarity—0.21). Halicin demonstrated efficacy against a majority of multidrug-resistant (MDR) pathogenic bacteria both in vitro and in vivo [14]. For instance, Higashihira et al. [17] indicated the effectiveness of halicin against the in vitro formation of biofilms by *S. aureus*. Zhao et al. [44] revealed that halicin, functioning as a JNK inhibitor, exhibited effective antifungal treatment outcomes at both the animal model and cellular levels. However, there is no evidence confirming the antibacterial effects of halicin on clinically isolated bacteria from animals. The in vitro antimicrobial activity of halicin versus six commonly used antimicrobial agents against 13 clinical bacterial strains was assessed through microbroth dilution (Table 1). Halicin exhibited a broad-spectrum activity, outperforming the commonly used antimicrobial agents (tetracycline, gentamicin, florfenicol, and trimethoprim–sulfamethoxazole) against *E. coli* (MIC_90_ = 16 µg/mL), multidrug-resistant *A. baumannii* (MIC_90_ = 16 µg/mL), *A. pleuropneumoniae* (MIC_90_ = 2 µg/mL)*, S. suis* (MIC_90_ = 16 µg/mL), *C. perfringens* (MIC_90_ = 0.25 µg/mL), and *Mycoplasma* (MIC_90_ = 1 µg/mL). Here, we demonstrated that compared to commonly used antimicrobial agents, halicin was a broad-spectrum antimicrobial agent and exhibited stronger antimicrobial activity against a variety of animal-derived pathogens, particularly *A. pleuropneumoniae*, *C. perfringens*, *S. suis*, and *Mycoplasma*. It should be noted that conducting long-term studies to evaluate the persistence of halicin’s antimicrobial action and to monitor the emergence of resistance during extended treatment periods is crucial, as the resistance monitoring programs are important for tracking resistance patterns and providing information for the management of antimicrobial drugs, which is essential for the comprehensive management of bacterial infections.

**Figure 1:**
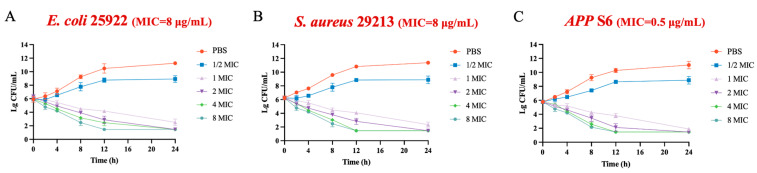
Antimicrobial kinetic curves of halicin against *E. coli* ATCC 25922 (**A**), *S. aureus* ATCC 29213 (**B**), and *APP* S6 (**C**).

Given the broad-spectrum antibacterial properties of halicin, we selected three classic bacterial strains, the Gram-negative bacterium *E. coli* ATCC 25922, the Gram-positive bacterium *S. aureus* ATCC 29213, and wild-type strain *APP* S6, for the further study on pharmacological and in vivo antimicrobial activities. The time-killing curve results illustrated a concentration-dependent bactericidal effect of halicin against *E. coli* ATCC 25922, *S. aureus* ATCC 29213, and *APP* S6 (Figure 1). For concentration-dependent antimicrobials, therapeutic effects could be enhanced by adjusting the dosage to achieve higher drug concentrations. This enables dose customization based on the severity of the infection, thereby providing increased flexibility in clinical settings.

### 3.2. The Antibacterial Characteristics of Halicin

**Figure 2:**
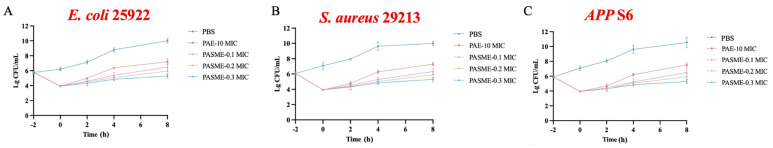
The post-antibiotic effects and post-antibiotic sub-minimum inhibitory concentration effects against *E. coli* ATCC 25922 (**A**), *S. aureus* ATCC 29213 (**B**), and *APP* S6 (C) for halicin.

Substantial growth inhibitions in PAE and PA-SME formats were attributed to nonlethal damage caused by antimicrobial agents and the subsequent recovery time from such damage [45,46,47]. To explore the potential for post-antibiotic effects (PAEs) and post-antibiotic sub-minimum inhibitory concentration effects (PA-SMEs) induced by halicin, ceftiofur was selected as the control for SU3327 against *E. coli* ATCC 25922, ciprofloxacin was used for *S. aureus* ATCC 29213, and ceftiofur was chosen for *APP* S6. Notably, halicin demonstrated prolonged PAE (>1 h) and PA-SME (>0.85 h) durations when compared to these control drugs (Figure 2 and Table 2). Halicin outperformed the control drugs in extending PAE and PA-SME effects, exhibiting a dose-responsive enhancement in the suppression of bacterial growth of *E. coli*, *S. aureus*, and *APP*, thereby underscoring its prolonged antimicrobial action beyond the immediate bacteriolytic impact at MIC levels. Odenholt et al. [48] reported that the PAE of teicoplanin against *S. aureus* ATCC 29213 was 1.0 h, while the PASMEs at 0.1, 0.2, and 0.3 × MIC were 1.6, 2.3, and 3.4 h, respectively. Additionally, Jacobs et al. [49] reported that the PAE of telithromycin against three strains of *S. aureus* was 0.3 to 2.4 h, with a PASME at concentrations of 0.12, 0.25, and 0.5 × MIC ranging from 1.9 to 2.4 h, 1.9 to 2.7 h, and 2.6 to 3.5 h, respectively. These results expand our comprehension of the antimicrobial activity of halicin beyond its potent immediate bacteriolytic effect at or above the MIC, indicating that halicin possessed robust in vivo antibacterial activity, with an efficacy superior or comparable to commonly used antimicrobial agents.

### 3.3. The Resistance Induction Profile of Halicin

**Figure 3:**
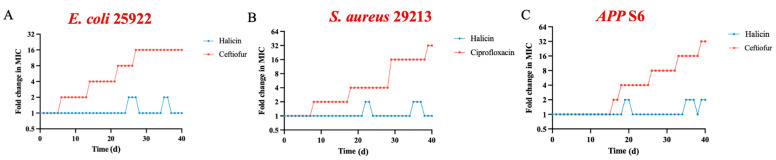
Evolution of resistance to halicin and control drugs in *E. coli* ATCC 25922 (**A**), *S. aureus* ATCC 29213 (**B**), and *APP* S6 (**C**) after 40 d of passaging.

To explore whether halicin is prone to inducing bacterial resistance, sub-minimum inhibitory concentrations (1/2 × MIC) were used to potentially induce resistance in *E. coli* ATCC 25922, *S. aureus* ATCC 29213, and *APP* S6. Results from the induced subculture assay (Figure 3A,B) revealed that after 40 passages, the MIC of halicin remained unchanged for *E. coli* ATCC 25922 (a 16-fold increase for ceftiofur) and *S. aureus* ATCC 29213 (a 32-fold increase for ciprofloxacin). The MIC for *APP* S6 increased from 0.5 µg/mL to 1 µg/mL (a 32-fold increase for ceftiofur) (Figure 3C), which indicated a lower propensity for halicin to induce bacterial resistance. This aligns with the results reported by Stokes [14]. Furthermore, the MPC of halicin for *E. coli* ATCC 25922, *S. aureus* ATCC 29213, and *APP* S6 indicated a relatively narrow MSW for halicin against these strains (Table 3), suggesting a reduced likelihood of resistance development. Levinson et al. [50] reported that the MPC and MSW of oxyclozanide against *S. aureus* were 16 µg/mL and 1–16 µg/mL, respectively. Pan et al. [51] reported that the MPC of fosfomycin against *E. coli* ATCC 25922 is 57.6 µg/mL, and the MSW ranges from 2 to 57.6 µg/mL. Dorey et al. [52] found that the MPC of oxytetracycline for isolated *A. pleuropneumoniae* was 44.4 µg/mL, with the MSW ranging from 2.5 to 44.4 µg/mL. In conclusion, the relatively narrow MSW of halicin, as well as the 40 d continuous drug induction assay, indicated its potential to mitigate resistance development.

### 3.4. Acute Toxicity Determination of Halicin in Mice

Here, we conducted the safety assessments of halicin through various toxicology assays. The intraperitoneal acute toxicity test in ICR mice revealed that the negative control group showed no toxicity, while halicin induced lethargy, ptosis, dilated pupils, and altered posture around 30 min post administration (Table 4 and Table S3). Deaths occurred approximately 50 min after treatment, and surviving mice gradually recovered within 30 h. The intraperitoneal mean lethal dose (LD_50_) of halicin via intraperitoneal administration in ICR mice was 36.84 mg/kg b.w, with a 95% confidence interval of 32.67 to 41.55 mg/kg b.w. In the oral acute toxicity assay (Table 4 and Table S4), most ICR mice showed symptoms within 20 min of receiving halicin. Mortality in the highest dose group occurred 50 min later, with death occurring between 1 to 24 h post administration. Surviving mice gradually recovered after 36 h. The oral LD_50_ of halicin in ICR mice was 1274.90 mg/kg b.w., with a 95% confidence interval of 1004.98 to 1617.32 mg/kg b.w. According to the Globally Harmonized System of Classification and Labeling of Chemicals (GHS), the LD_50_ of halicin was categorized into Category 4, defined by the range between 300 and 2000 mg/kg b.w. [53]. Antimicrobial drugs including ofloxacin, amikacin, macleaya cordata extract, and nitazoxanide are also classified under GHS Category 5 (>2000 mg/kg b.w.), 5, 5, 4, and 4, respectively [54,55,56,57]. Post-mortem examination of both deceased and surviving mice in all dose groups revealed no abnormalities in organs in both intraperitoneal and oral administration acute toxicity tests.

The Ames test, extensively applied to assess mutagenic and carcinogenic risks, has identified a total of 5000 chemical compounds with the risks [58,59]. The potential reproductive toxicity and mutagenicity of compounds can be evaluated through the mouse sperm abnormality test, chromosomal aberration test in bone marrow cells, and bone marrow micronucleus test [60,61]. In this study, the Ames test results for halicin (Table 4, Tables S5 and S6), conducted in the range from 0.0004176 μg/plate to 0.261 μg/plate with or without the metabolic activation system (S9), consistently showed no statistically significant differences (*p* > 0.05) in average revertant colonies per plate compared to the negative control group for all *Salmonella typhimurium* strains. This indicated a negative outcome for the *Salmonella typhimurium* reverse mutation test, suggesting that halicin did not possess mutagenic properties on the tested strains. The mouse sperm abnormality test, conducted after the oral administration of halicin in the dose range of 39.84 to 159.36 mg/kg b.w., revealed no statistically significant differences in sperm abnormality rates compared to the negative control group (*p* > 0.05, Table 4 and Table S7). Therefore, halicin was considered negative for mouse sperm abnormality. In addition, both the mouse bone marrow micronucleus test (Table 4, Tables S8 and S9) and the chromosomal aberration test (Table 4, Tables S10 and S11), carried out with oral doses ranging from 159.36 to 637.45 mg/kg b.w., indicated no significant variances in micro-nucleated polychromatic erythrocyte rates or chromosomal aberration rates when compared to the negative control group (*p* > 0.05). Thus, both tests yielded negative results for halicin. It is said that compounds containing nitro groups might have mutagenic potential [62]. Fei et al. evaluated the safety of nitromezuril through tests including bacterial reverse mutation, sperm abnormalities, micronuclei, and chromosomal aberrations, and revealed that nitromezuril did not induce mutations in strains TA_97_ and TA_1535_ at any concentration, regardless of the presence of the S9 mix. However, it did cause significant mutations in strains TA_98_ and TA_100_. In contrast, structurally related drugs, such as diclazuril and toltrazuril, yielded negative results in these tests. Additionally, nitromezuril demonstrated a high level of safety in the mouse sperm abnormality test, chromosomal aberration test in bone marrow cells, and bone marrow micronucleus test [63]. In conclusion, halicin exhibited lower acute toxicity, being non-mutagenic, non-reproductive, and non-genotoxic. However, future studies on subchronic and chronic toxicological data for halicin should be performed to comprehensively assess its safety.

### 3.5. In Vivo Toxicity Assessment of Halicin

**Figure 4:**
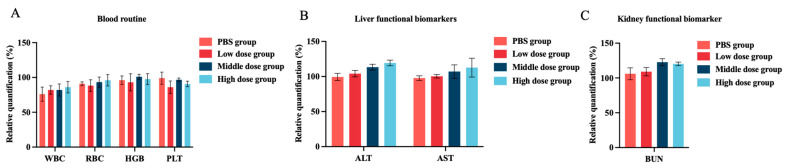
In vivo toxicity of halicin of oral administration for 48 h. Changes in the hematological parameters of (**A**) blood routine; (**B**) liver functional biomarkers; and (**C**) kidney functional biomarker in mice followed by the oral administration of halicin (the high-, medium-, and low-dose groups were 25.48, 12.74, and 6.37 mg/kg b.w, respectively).

**Figure 5:**
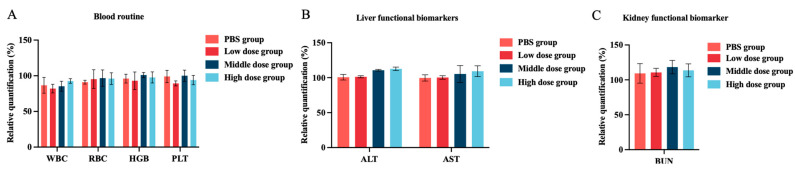
In vivo toxicity of halicin of intraperitoneal injection for 48 h. Changes in the hematological parameters of (**A**) blood routine; (**B**) liver functional biomarkers; and (**C**) kidney functional biomarker in mice followed by the oral administration of halicin (the high-, medium-, and low-dose groups were 3.69, 1.47, and 0.74 mg/kg b.w., respectively).

Reports on the in vivo safety of halicin were limited. Hussain et al. [64] showed that the combined administration of halicin and doxycycline did not significantly affect the heart, liver, spleen, lung, or kidney in mice by H&E staining, indicating no tissue or organ damage. Additionally, there were no significant changes in blood biochemical parameters (WBC, RBC, HGB, and PLT), liver function biomarkers (ALT and AST), or kidney function biomarkers (Creatinine and BUN), suggesting a high safety profile for halicin. To comprehensively evaluate the in vivo toxicity of halicin in mice, various doses of halicin (25.48, 12.74 and 6.37 mg/kg b.w.) were orally administrated. Blood samples were collected and analyzed 48 h post administration. The results showed no statistically significant changes in blood parameters (WBC, RBC, HGB, and PLT) (Figure 4A). Similarly, no significant alterations were observed in liver function biomarkers (ALT and AST) and kidney function biomarkers (BUN) (Figure 4B,C). Additionally, the intraperitoneal injection of different doses (3.69, 1.47, and 0.74 mg/kg b.w.) did not result in significant changes in blood routine, liver function, and kidney function (Figure 5A–C). The histopathological examination of the lung, heart, spleen, kidney, and liver revealed no substantial pathological changes compared to the vehicle and control groups (Figure S1). These findings are consistent with those reported by Hussain [64]. Overall, the administration of halicin demonstrated minimal or no systematic toxicity. Furthermore, the use of halicin in veterinary clinics should take into account its potential toxicity. It is advisable to employ lower doses that are still within the effective therapeutic range or to improve safety by combining it with other drugs.

### 3.6. In Vivo Antimicrobial Activity Study of Halicin

**Figure 6:**
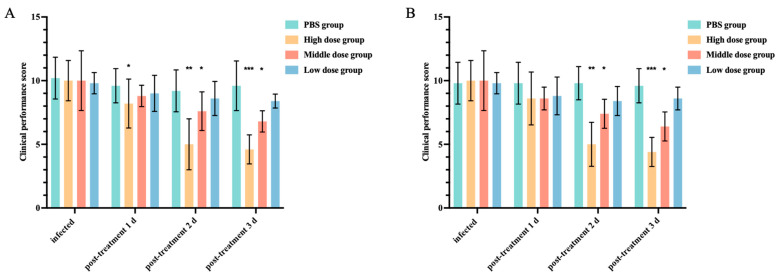
The influence of halicin with oral administration (**A**) (the high-, medium-, and low-dose groups were 25.48, 12.74, and 6.37 mg/kg b.w., respectively) and intraperitoneal injection (**B**) (the high-, medium-, and low-dose groups were 3.69, 1.47, and 0.74 mg/kg b.w., respectively) on clinical symptom scores in murine respiratory infection with *APP* S6. The data is represented as mean ± SEM, with six biological replicates per group. One-way ANOVA was used to calculate *p*-values (* *p* < 0.05, ** *p* < 0.01, *** *p* < 0.001).

**Figure 7:**
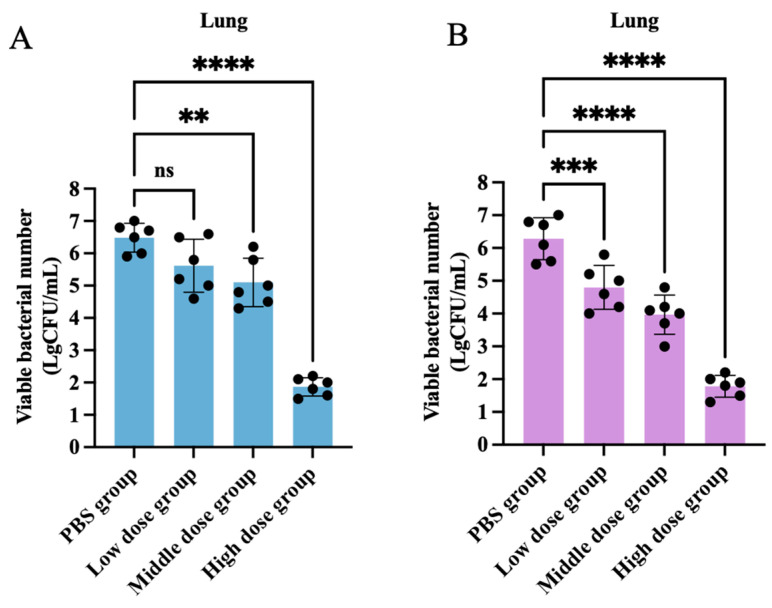
Changes in lung bacterial load after 48 h of oral administration (**A**) (the high-, medium-, and low-dose groups were 25.48, 12.74, and 6.37 mg/kg b.w., respectively) and intraperitoneal injection (**B**) (the high-, medium-, and low-dose groups were 3.69, 1.47, and 0.74 mg/kg b.w., respectively) with halicin (mg/kg b.w.) against *APP* S6 in mouse respiratory infection models. The data is represented as mean ± SEM, with six biological replicates per group. One-way ANOVA was used to calculate *p*-values (** *p* < 0.01, *** *p* < 0.001, **** *p* < 0.0001).

**Figure 8:**
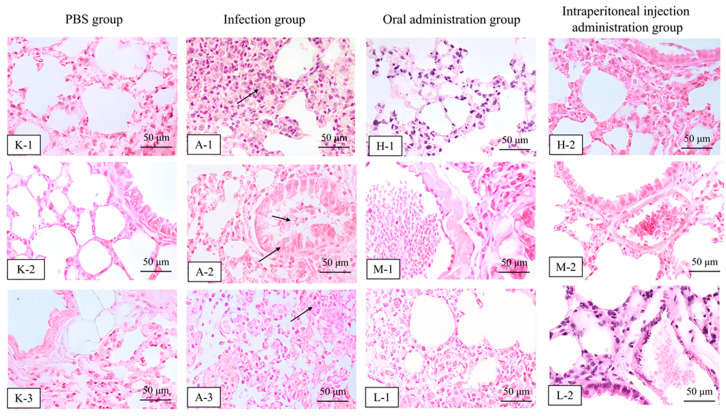
Histopathological analysis in mice in the PBS group (K–1, K–2 and K–3), infected group (A–1, A–2 and A–3), oral (the H–1, M–1, and L–1 were 25.48, 12.74, and 6.37 mg/kg b.w., respectively) and intraperitoneal administration 48 h after treatment with halicin (the H–2, M–2, and L–2 were 3.69, 1.47, and 0.74 mg/kg b.w., respectively) evaluated by H&E staining (100×, scale bar: 50 μm). Note: the arrows in the images indicate varying degrees of pathological changes (pulmonary congestion, lung hemorrhage, and inflammatory cell infiltration) in the lung tissue after infection with *APP* S6 in mice.

In our study, we demonstrated that halicin exhibited strong in vitro antibacterial activity against *A. pleuropneumoniae*, with an MIC_90_ value of 2 μg/mL, and revealed a low propensity for inducing antibiotic resistance. These findings suggest the potential of halicin as a novel antibacterial agent for the treatment of *A. pleuropneumoniae* infections. In this study, we employed a murine model of respiratory tract infection induced by *APP* S6. A period of 24 h post infection, the *APP* S6 strain could be detected in the blood, heart, liver, spleen, lungs, and kidneys of mice. Halicin exhibited favorable therapeutic effects in combating infections of the respiratory tract caused by *APP* S6. Notably, after infection with *APP* S6, animals treated with halicin showed a marked reduction in clinical symptom scores on the 2nd and 3rd day post treatment, via both oral and intraperitoneal administration routes, particularly within the high-dose and medium-dose groups (Figure 6). Halicin exhibited significant bacterial load clearance capabilities compared to the PBS (0.1 M) group (*p* < 0.05) (Figure 7 and Figure S2). Furthermore, the lung tissue of the infection group exhibited alveolar wall thickening accompanied by inflammatory cell infiltration. Epithelial shedding occurred in the bronchioles, and the vascular lumens were congested and filled with inflammatory cells. In the halicin treatment group, although vascular congestion was present, the morphology and structure of the alveolar walls were regular, indicating its potential as a therapeutic agent (Figure 8 and Figure S3). The mouse model for respiratory infections demonstrated that halicin, administered orally or via intraperitoneal injection, exhibited a dose-dependent reduction in bacterial load. Histopathological examination further substantiated that both modes of halicin administration effectively mitigated inflammatory responses, highlighting its in vivo efficacy in the treatment of respiratory tract infections induced by *A. pleuropneumoniae*. Numerous studies have explored the antibacterial effects of plant extracts; particularly, phenols and flavonoids have demonstrated their effectiveness [65]. Ding et al. [66] administered Rhein intraperitoneally at a dose of 80 mg/kg b.w. in mice infected with *A. pleuropneumoniae*, which significantly reduced the bacterial load in the lungs and substantially alleviated lung damage. The research by Wang et al. [67] showed that thymol administered intraperitoneally at a dose of 20 mg/kg b.w. could protect mice from lethal infections of *A. pleuropneumoniae* and reduce pulmonary pathological lesions. In this study, halicin demonstrated inhibitory activity when administered orally in an infected mouse model, which suggest that halicin had survived the liver metabolism, degradation by stomach acids and digestive enzymes, and absorption interference from the digestive contents of the treated mice [68]. In addition, halicin can be effective at lower doses in the treatment of respiratory infections when administered intraperitoneally. These results suggest that halicin holds promising potential for application in veterinary clinical settings against *A. pleuropneumoniae* infections. Further pharmaceutical and pharmacodynamic studies in mice and swine are required.

### 3.7. Clinical Significance, Limitations, and Future Prospects of Halicin

Halicin, a novel antimicrobial agent, demonstrated broad-spectrum antibacterial activity against various animal pathogens, while featuring low toxicity and a reduced tendency to induce resistance. These characteristics not only offer new therapeutic options for infections from veterinary pathogens but also facilitate the control and prevention of the spread of resistant strains. Furthermore, these properties underscore the significance of the “One Health” concept, highlighting the need for long-term monitoring and research on halicin. When formulating public health policies, it is also crucial to consider halicin’s potential impacts on the environment and ecology, which could promote a holistic and sustainable approach to health across humans, animals, and the environment [5,69]. However, there are some limitations, including the lack of long-term subchronic and chronic toxicity assessments, inadequate studies on resistance development, absence of clinical application data, limited information on pharmacokinetics and metabolism, and insufficient efficacy evaluations in diverse bacterial infection models. These gaps point to the directions for future research to establish a more comprehensive and robust scientific basis for halicin’s clinical application.

Concerning the antimicrobial mechanism of halicin, Stokes et al. [14] demonstrated through transcriptomic analysis that halicin could disrupt the △pH component of the bacterial respiratory chain’s proton motive force (PMF), suggesting that its mechanism of action is related to the respiratory chain. Additionally, the action mechanism of nitro-group-containing drugs may involve bacterial DNA damage, since the nitro group can react with bacterial nitroreductases to form substances that cause DNA damage [70,71,72]. Therefore, we hypothesize that the antimicrobial target of halicin may be related to bacterial DNA.

## 4. Conclusions

Halicin has demonstrated broad-spectrum antibacterial efficacy, particularly against respiratory infections caused by animal-derived pathogen *A. pleuropneumoniae* in mouse models. Its low-toxicity profile enhances its potential for clinical application, promising a new direction in the treatment of bacterial infections resistant to currently used antimicrobial agents. Consequently, halicin emerges as a promising candidate for further research and development aimed at veterinary clinical applications.

## Figures and Tables

**Table 1:** Determination of minimum inhibitory concentration and minimum bactericidal concentration of SU3327 and commonly used antimicrobial agents against the test pathogens (20 strains each, totaling 260 strains).

Pathogenic Bacteria	MIC_90_ (µg/mL)							MBC_90_ (µg/mL)						
	HAL	CEF	GEN	TET	CIP	FLO	SMZ-TMP	HAL	CEF	GEN	TET	CIP	FLO	SMZ-TMP
*Escherichia coli*	16	≤1	16	>128	16	128	>121.6:6.4	32	2	64	>128	32	>128	>121.6:6.4
*Salmonella*	16	>256	64	>256	32	>256	>243.2:12.8	64	>256	256	256	64	>256	243.2:12.8
*Klebsiella penumoniae*	16	32	32	16	16	16	32	64	64	128	64	64	128	60.8:3.2
*Staphylococcus* spp.	16	8	32	16	8	8	30.4:1.6	64	16	64	64	32	32	60.8:3.2
*Streptococcus* spp.	16	≤1	>128	32	≤1	2	>121.6:6.4	128	≤1	>128	>128	>4	>8	>121.6:6.4
*Streptococcus suis*	16	4	>128	>128	32	128	>121.6:6.4	32	64	>128	>128	64	>128	>121.6:6.4
*Pasteurella multocida*	8	>32	1	>32	0.25	>32	>30.4:1.6	8	>32	4	>32	>1	>32	>30.4:1.6
*Actinobacillus pleuropneumoniae*	2	8	4	>32	4	>32	>30.4:1.6	4	8	4	>32	>32	>32	>30.4:1.6
*Haemophilus parasuis*	16	≤0.5	≤0.5	8	1	≤0.5	60.8:3.2	128	≤0.5	1	16	>2	2	>60.8:3.2
*Clostridium perfringens*	0.25	≤0.125	>16	>16	>16	2	>15.2:0.8	0.5	1	>16	>16	>16	>8	>15.2:0.8
*Mycoplasma*	1	>128	4	4	2	4	3.8:0.2	1	>128	8	8	8	16	7.6:0.4
*Pseudomonas aeruginosa*	>128	32	32	64	32	32	30.4:1.6	>128	128	128	64	64	32	60.8:3.2
*Acinetobacter baumannii*	16	8	16	8	8	8	30.4:1.6	64	32	32	32	32	32	60.8:3.2

Note: HAL = halicin; CEF = ceftiofur; GEN = gentamicin; TET = tetracycline; CIP = ciprofloxacin; FLO = florfenicol; SMZ-TMP = trimethoprim–sulfamethoxazole.

**Table 2:** Comparison of the post-antibiotic effects and post-antibiotic sub-minimum inhibitory concentration effects of antibacterial drugs against *E. coli* ATCC 25922, *S. aureus* ATCC 29213, and *APP* S6.

Strain	Antibacteri-al Drugs	MIC (μg/mL)	MBC (μg/mL)	PAE (h)	PASME (h)		
					0.1 MIC	0.2 MIC	0.3 MIC
*E. coli* 25922	Halicin	8	16	1.52	2.03	2.43	3.05
	Ceftiofur	1	4	0.89	1.23	1.56	1.78
*S. aureus* 29213	Halicin	8	16	1.45	1.89	2.56	3.24
	Ciprofloxacin	1	2	1.23	1.45	1.75	1.98
*APP* S6	Halicin	1	2	1.03	0.85	1.08	1.63
	Ceftiofur	2	8	0.68	0.45	0.87	1.25

Note: “h” represents hour.

**Table 3:** The minimum preventable concentrations and mutant selection windows for halicin and control drugs against *E. coli* ATCC 25922, *S. aureus* ATCC 29213, and *APP* S6.

Strains	Antibacterial Drugs	MIC (μg/mL)	MPC (μg/mL)	MSW (μg/mL)
*E. coli* 25922	Halicin	8	12.8	8–12.8
	Ceftiofur	2	12.8	2–12.8
*S. aureus* 29213	Halicin	8	12.8	8–12.8
	Ciprofloxacin	4	25.6	4–25.6
*APP* S6	Halicin	0.5	1.6	0.5–1.6
	Ceftiofur	1	6.4	1–6.4

**Table 4:** Toxicological test results of halicin.

Experiments	Results
Oral acute toxicity test	LD_50_ was 1274.90 mg/kg b.w.
Intraperitoneal injection acute toxicity test	LD_50_ was 36.84 mg/kg b.w.
Ames test	No significant difference in the average number of the four *Salmonella typhimurium* test strains compared to the negative control group
Mouse sperm abnormality test	No significant difference between the dose group and the negative control group (*p* > 0.05)
Chromosomal aberration test in bone marrow cells	No significant difference between the dose group and the negative control group (*p* > 0.05)
Bone marrow micronucleus test	No significant difference between the dose group and the negative control group (*p* > 0.05)

## Data Availability

Source data supporting the findings of the present study are included in the article and Supplementary Materials.

## Supplementary Materials

The following supporting information can be downloaded at https://www.mdpi.com/article/10.3390/antibiotics13060492/s1, Table S1: Observation parameters of intoxication symptoms in mice; Table S2: Clinical symptom scoring criteria for respiratory tract infection; Table S3: Mortality statistics for each dosage group; Table S4: Mortality statistics for each dosage group; Table S5: Number of revertant colonies in the first trial of the *Salmonella typhimurium* reverse mutation test ($\bar{X}$ ± SD); Table S6: Number of revertant colonies in the second trial of the *Salmonella typhimurium* reverse mutation test ($\bar{X}$ ± SD); Table S7: Results of the sperm morphology test in mice (Sperm abnormality rate); Table S8: Results of the micronucleus assay of mouse bone marrow cells in mice (Female mice); Table S9: Results of the micronucleus assay of mouse bone marrow cells in mice (Male mice); Table S10: Results of the chromosomal aberration test of bone marrow cells in mice (Female mice); Table S11: Results of the chromosomal aberration test of bone marrow cells in mice (Male mice); Figure S1: The histopathological analysis of heart, liver, spleen, lung, and kidney emulated by H&E staining in different groups after 48 h with oral administration (A) (The high, medium, and low dose groups were 25.48, 12.74, and 6.37 mg/kg b.w, respectively.) and intraperitoneal injection (B) of halicin (The high, medium, and low dose groups were 3.69, 1.47, and 0.74 mg/kg b.w, respectively.) (40×, Scale bar:100 μm); Figure S2: Bacterial load changes in blood, heart, liver, spleen, lung and liver after 24 h of oral administration (A) (The high, medium, and low dose groups were 25.48, 12.74, and 6.37 mg/kg b.w, respectively.) and intraperitoneal injection (B) (The high, medium, and low dose groups were 3.69, 1.47, and 0.74 mg/kg b.w, respectively.) with halicin (mg/kg) against *APP* S6 in mouse respiratory infection model; Figure S3: Histopathological analysis in infected mice 48 h after oral (The H1, M1, L1 were 25.48, 12.74, and 6.37 mg/kg b.w., respectively) or intraperitoneal administration (The H2, M2, L2 were 3.69, 1.47, and 0.74 mg/kg b.w., respectively) of halicin evaluated by H&E staining (40×, Scale bar: 100 μm).
