# Supplementary material for: Halicin: A New Horizon in Antibacterial Therapy against Veterinary Pathogens
Source: Antibiotics (Basel). 2024 May 27;13(6):492. doi: 10.3390/antibiotics13060492 (PMC11200678; doi:10.3390/antibiotics13060492)
Supplement: Supplementary file 1 [file antibiotics-13-00492-s001.zip › antibiotics-2993544-supplementary.pdf]

Supplemental material

Table S1. Observation parameters of intoxication symptoms in mice

Organ systems	Observation and examination items	General manifestations post-intoxication
Central nervous system and motor activity	Behavior	Altered posture, abnormal vocalizations, restlessness, stupor
	Action	Tremors, ataxia, paralysis, rigidity
	Various stimulation reactions	Hyperactivity, heightened sensitivity, or altered perception
	Brain and spinal cord reflexes	Reduced or absent
	Muscle tone	Stiffness, slowness
Autonomic nervous system	Pupil size	Constriction or dilation
	Secretions	Drooling, tearing
Respiratory system	Nasal cavity	Nasal discharge
	Respiratory character and respiratory rate	Slow, difficult, cheyne-stokes respiration
Gastrointestinal system	Abdomen	Bloating or contraction, diarrhea or
	Fecal consistency and color	Loose feces, black or grey
Reproductive and urinary system	Vulva, Mammary gland	Distension
	Penis	Prolapse
	Perineum	Dirty
Skin and Coat	Color, Tension	Reddened, wrinkled, flaccid, or rash
	Integrity	Piloerection
Mucous Membranes	Mucosa	Mucous discharge, congestion, hemorrhagic cyanosis, or pale
	Oral cavity	Ulcer
Eye	Eyelid	Drooping upper eyelid or ptosis
	Eyeball	Proptosis or ocular tremor
	Transparency	Turbidity
Others	Rectal temperature or Skin temperature	Decrease or increase
	General condition	Abnormal posture, emaciation

Table S2. Clinical symptom scoring criteria for respiratory tract infection

	Appetite	Respiratory Rate	Mental state	Nasal secretions
0	Normal	Normal	Normal	No
1	Slight reduction	Slight increase	Slight malaise	Nasal discharge
2	Significant reduction	Significant increase	Lethargic	Significant elevation
3	Severe decline or anorexia	Shortness of breath with wheezing	Somnolence, lethargy, and slowed responsiveness	Runny nose with excessive nasal discharge

Table S3. Mortality statistics for each dosage group.

Dosage group (mg/kg b.w.)	Total	Death	Mortality rate (%)	Survival rate (%)
80.00	10	10	100	0
60.65	10	10	100	0
45.98	10	8	80	20
34.86	10	4	40	60
26.43	10	1	10	90
20.04	10	0	0	100

Table S4. Mortality statistics for each dosage group

Dosage group (mg/kg b.w.)	Total	Death	Mortality rate (%)	Survival rate (%)
5000.00	10	10	100	0
3170.58	10	9	90	10
2010.51	10	8	80	20
1274.90	10	5	50	50
808.43	10	3	30	70
512.64	10	0	0	100

Table S5. Number of revertant colonies in the first trial of the *Salmonella typhimurium* reverse mutation test ($\overline{X} \pm SD$)

Groups	TA_{97}		TA_{98}		TA_{100}		TA_{102}	
	+ S9	- S9	+ S9	- S9	+ S9	- S9	+ S9	- S9
0.261 μg	127.3 ± 14.0	132.7 ± 14.7	36.3 ± 5.5	45.0 ± 6.1	181.3 ± 5.0	178.3 ± 11.5	281.0 ± 9.2	274.0 ± 7.2
0.0522 μg	128.0 ± 16.4	134.3 ± 13.3	38.7 ± 4.9	39.0 ± 9.5	178.0 ± 14.8	171.7 ± 8.3	278.7 ± 9.0	287.3 ± 10.4
0.01044μg	124.0 ± 15.0	139.7 ± 4.9	44.7 ± 6.7	39.7 ± 5.7	171.3 ± 13.8	180.3 ± 1.5	287.3 ± 10.8	274.3 ± 11.0
0.002088μg	135.7 ± 4.2	129.0 ± 15.9	35.3 ± 5.8	41.3 ± 6.0	174.7 ± 12.7	167.0 ± 4.6	280.3 ± 11.2	273.3 ± 13.7
0.0004176μg	131.3 ± 16.5	127.3 ± 9.5	42.7 ± 9.5	36.0 ± 3.5	170.3 ± 5.9	174.7 ± 15.0	281.0 ± 3.6	278.7 ± 13.6
Negative control 1	143.3 ± 5.5	133.7 ± 8.3	42.0 ± 6.0	38.0 ± 5.2	175.3 ± 10.5	176.3 ± 10.8	286.0 ± 19.9	273.0 ± 12.1
Negative control 2	136.3 ± 4.7	136.0 ± 7.2	38.3 ± 8.5	42.0 ± 9.5	182.0 ± 7.0	171.7 ± 7.6	286.3 ± 11.1	270.3 ± 17.0
Positive control	889.3 ± 42.8*	944.0 ± 17.4*	602.7 ± 10.1*	693.3 ± 26.6*	910.7 ± 31.1*	1070.3 ± 36.5*	1185.3 ± 40.5*	1246.7±54.5*
	2-AF (10 μg)	Fenaminosulf (50 μg)	2-AF (10 μg)	Fenaminosulf (50 μg)	2-AF (10 μg)	Sodium azide (1.5 μg)	2-AF (10 μg)	Fenaminosulf (50 μg)

Note: Negative control 1—sterile deionized water; Negative control 2—DMSO; Sodium azide—NaN_3; 2-AF—2-aminofluorene; positive control doses are in parentheses (μg/plate). Reversion colony count differences were statistically analyzed (t-test) within each column. The colony counts of revertants in each dose group of the test substance and the sodium azide positive control group were compared with the sterile deionized water group (Negative control 1). For other positive control groups, the colony counts were compared with the DMSO group (Negative control 2). Significant differences ($P < 0.05$) are marked with an asterisk (*), while non-significant differences ($P > 0.05$) are indicated without an asterisk.

Table S6. Number of revertant colonies in the second trial of the *Salmonella typhimurium* reverse mutation test ($\overline{X}$ ± SD)

Groups	TA_{97}		TA_{98}		TA_{100}		TA_{102}	
	+ S9	- S9	+ S9	- S9	+ S9	- S9	+ S9	- S9
0.261μg	126.0 ± 11.8	123.7 ± 6.8	40.3 ± 7.2	37.3 ± 5.9	179.0 ± 6.6	176.0 ± 12.5	276.7 ± 11.7	280.0 ± 14.7
0.0522μg	122.3 ± 9.7	117.3 ± 13.3	44.7 ± 6.7	37.7 ± 4.0	173.7 ± 11.7	170.7 ± 13.6	279.3 ± 15.0	277.0 ± 7.0
0.01044μg	131.7 ± 12.1	131.0 ± 14.7	40.7 ± 4.7	47.0 ± 3.0	171.7 ± 9.0	171.3 ± 9.1	267.0 ± 6.2	283.3 ± 9.5
0.002088μg	120.3 ± 11.5	133.7 ± 15.4	38.7 ± 4.7	40.3 ± 9.1	174.7 ± 11.7	178.7 ± 4.5	272.0 ± 10.6	275.7 ± 4.7
0.0004176μg	127.7 ± 9.0	128.0 ± 9.0	41.0 ± 8.5	42.0 ± 8.7	170.3 ± 11.9	176.3 ± 1.2	280.7 ± 5.1	283.3 ± 14.0
Negative control 1	123.7 ± 13.6	121.3 ± 7.5	38.3 ± 8.5	43.3 ± 4.5	180.7 ± 6.7	179.0 ± 9.0	280.7 ± 12.1	278.0 ± 15.5
Negative control 2	127.7 ± 4.0	127.0 ± 9.6	36.3 ± 6.8	39.0 ± 8.7	172.0 ± 8.5	177.0 ± 13.5	273.3 ± 16.3	271.7 ± 9.1
Positive control	910.7 ± 16.7*	768.0 ± 8.0*	622.7 ± 46.0*	628.0 ± 16.0*	837.3 ± 18.9*	978.7 ± 61.2*	1096.0 ± 35.6*	1128.0 ± 50.0*
	2-AF (10 μg)	Fenaminosulf (50 μg)	2-AF (10 μg)	Fenaminosulf (50 μg)	2-AF (10 μg)	Sodium azide (1.5 μg)	2-AF (10 μg)	Fenaminosulf (50 μg)

Note: Negative control 1—sterile deionized water; Negative control 2—DMSO; Sodium azide—NaN_3; 2-AF—2-aminofluorene; positive control doses are in parentheses (μg/plate). Reversion colony count differences were statistically analyzed (t-test) within each column. The colony counts of revertants in each dose group of the test substance and the sodium azide positive control group were compared with the sterile deionized water group (Negative control 1). For other positive control groups, the colony counts were compared with the DMSO group (Negative control 2). Significant differences ($P < 0.05$) are marked with an asterisk (*), while non-significant differences ($P > 0.05$) are indicated without an asterisk.

Table S7. Results of the sperm morphology test in mice (Sperm abnormality rate)

Groups	Number of animals	Sperm examination count	Abnormal sperm count	Sperm abnormality rate (%)	Statistical significance
High-dose group	5	5 × 1000	150	2.99 ± 0.81	$P > 0.05$
Middle-dose group	5	5 × 1000	134	2.68 ± 0.69	$P > 0.05$
Low-dose group	5	5 × 1000	145	2.90 ± 0.74	$P > 0.05$
Negative control	5	5 × 1000	140	2.80 ± 0.63	—
Positive control	5	5 × 1000	298	6.02 ± 0.84	$P < 0.01$

Note: Statistical significance refers to the comparison of sperm abnormality rates in each dose group and the positive control group with the negative (solvent) control in mice.

Table S8. Results of the micronucleus assay of mouse bone marrow cells in mice (Female mice)

Groups	Dose (mg/kg b.w.)	Number of animals	PCE/RBC ratio	PCE micronucleus rate (‰)	Statistical significance
High-dose group	637.45	5	0.97 ± 0.08	4.60 ± 1.76	$P > 0.05$
Middle-dose group	318.73	5	0.95 ± 0.06	5.20 ± 1.87	$P > 0.05$
Low-dose group	159.36	5	0.96 ± 0.05	5.46 ± 1.94	$P > 0.05$
Negative control	—	5	0.90 ± 0.08	5.34 ± 1.99	—
Positive control	40.00	5	0.86 ± 0.14	21.66 ± 5.80	$P < 0.01$

Note: Statistical significance (X^2 test) refers to the comparison of PCE (polychromatic erythrocyte) micronucleus rates in each dose group and the positive control group with the negative control group.

Table S9. Results of the micronucleus assay of mouse bone marrow cells in mice (Male mice)

Groups	Dose (mg/kg b.w.)	Number of animals	PCE/RBC ratio	PCE micronucleus rate (‰)	Statistical significance
High-dose group	637.45	5	0.92 ± 0.10	5.29 ± 1.84	$P > 0.05$
Middle-dose group	318.73	5	0.94 ± 0.05	4.55 ± 2.34	$P > 0.05$
Low-dose group	159.36	5	0.94 ± 0.08	4.59 ± 1.44	$P > 0.05$
Negative control	—	5	0.93 ± 0.09	4.77 ± 1.78	—
Positive control	40.00	5	0.77 ± 0.08	19.00 ± 4.50	$P < 0.01$

Note: Statistical significance (X^2 test) refers to the comparison of PCE (polychromatic erythrocyte) micronucleus rates in each dose group and the positive control group with the negative control group.

Table S10. Results of the chromosomal aberration test of bone marrow cells in mice (Female mice)

Groups	Dose (mg/kg b.w.)	Number of cells examined in the	Number of animals	Number of cells with chromosomal aberrations	Aberration rate (%)	Statistical significance
High-dose group	637.45	5 × 100	5	5	1.00 ± 0.45	$P > 0.05$
Middle-dose group	318.73	5 × 100	5	5	1.00 ± 0.42	$P > 0.05$
Low-dose group	159.36	5 × 100	5	7	1.40 ± 0.47	$P > 0.05$
Negative control	—	5 × 100	5	7	1.40 ± 0.50	—
Positive control	40.00	5 × 100	5	134	26.80 ± 1.80	$P < 0.01$

Note: Statistical significance refers to the comparison of the chromosomal aberration cell rates in each dose group and the positive control group with the negative (solvent) control in mice.

Table S11. Results of the chromosomal aberration test of bone marrow cells in mice (Male mice)

Groups	Dose (mg/kg b.w.)	Number of cells examined in the mid-term phase	Number of animals	Number of cells with chromosomal aberrations	Aberration rate (%)	Statistical significance
High-dose group	637.45	5 × 100	5	6	1.20 ± 0.47	$P > 0.05$
Middle-dose group	318.73	5 × 100	5	7	1.40 ± 0.47	$P > 0.05$
Low-dose group	159.36	5 × 100	5	6	1.20 ± 0.48	$P > 0.05$
Negative control	—	5 × 100	5	5	1.00 ± 0.45	—
Positive control	40.00	5 × 100	5	144	28.80 ± 1.43	$P < 0.01$

Note: Statistical significance refers to the comparison of the chromosomal aberration cell rates in each dose group and the positive control group with the negative (solvent) control in mice.

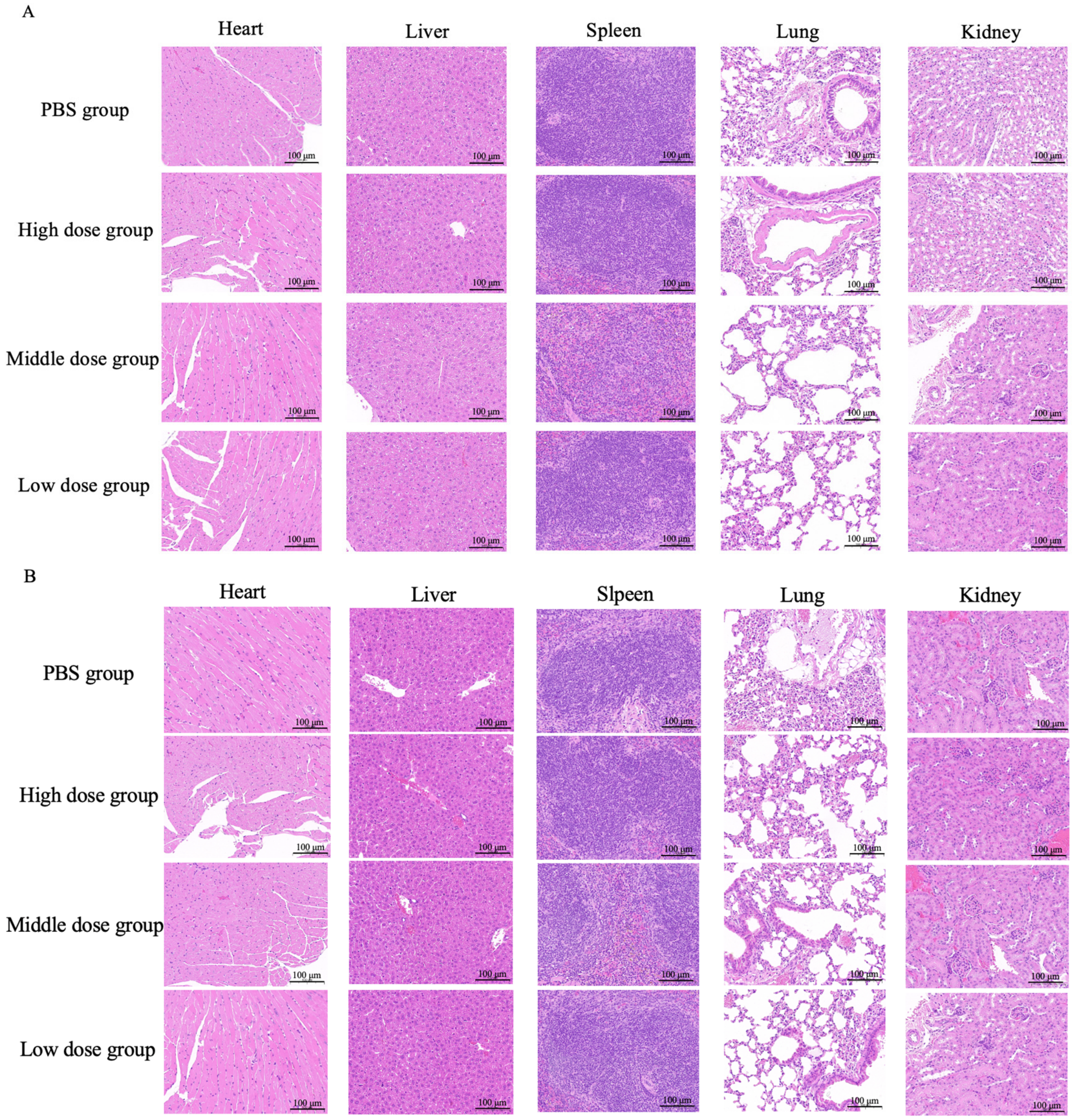

Figure S1. The histopathological analysis of heart, liver, spleen, lung, and kidney emulated by H&E staining in different groups after 48 h with oral administration (A) (The high, medium, and low dose groups were 25.48, 12.74, and 6.37 mg/kg b.w, respectively.) and intraperitoneal injection (B) of halicin (The high, medium, and low dose groups were 3.69, 1.47, and 0.74 mg/kg b.w, respectively.) (40×, Scale bar:100 μm)

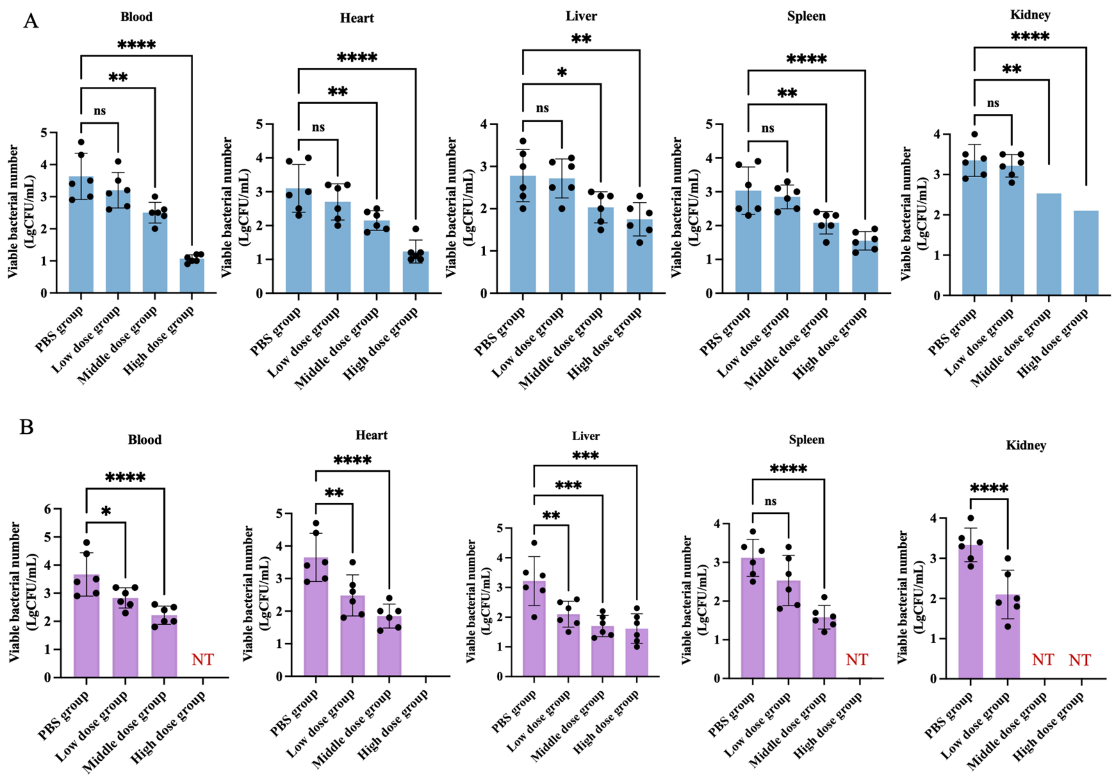

Note: “NT” represents “not detected”.

Figure S2. Bacterial load changes in blood, heart, liver, spleen, lung and liver after 24 h of oral administration (A) (The high, medium, and low dose groups were 25.48, 12.74, and 6.37 mg/kg b.w, respectively.) and intraperitoneal injection (B) (The high, medium, and low dose groups were 3.69, 1.47, and 0.74 mg/kg b.w, respectively.) with halicin (mg/kg) against *APP* S6 in mouse respiratory infection model

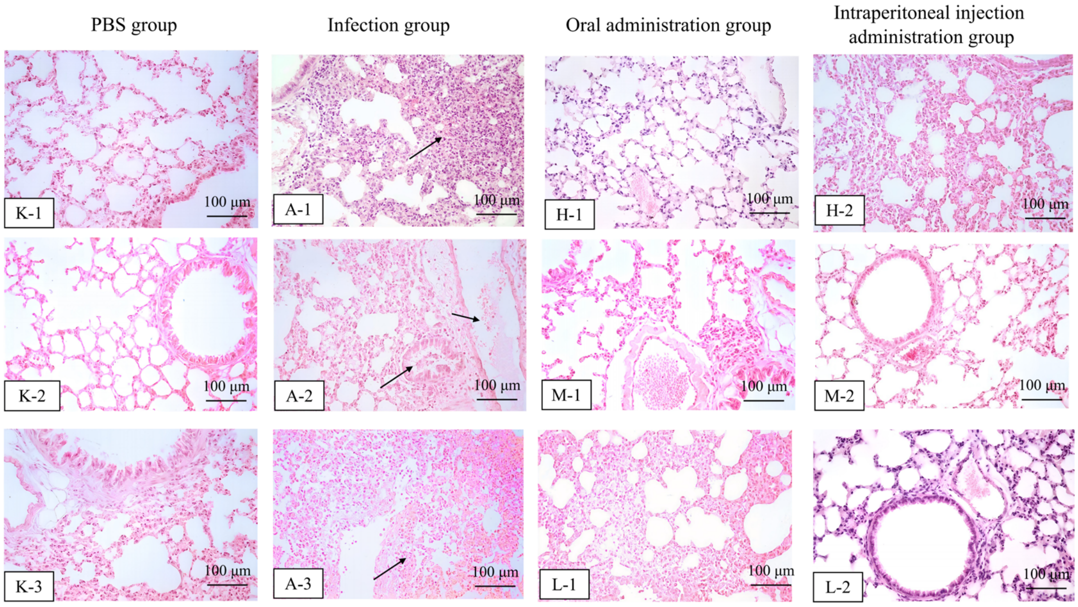

Note: The arrows in the image indicated varying degrees of pathological changes (pulmonary congestion, lung haemorrhage and inflammatory cell infiltration) in the lung tissue after infected with *APP* S6 in mice.

Figure S3. Histopathological analysis in infected mice 48 h after oral (The H1, M1, L1 were 25.48, 12.74, and 6.37 mg/kg b.w., respectively) or intraperitoneal administration (The H2, M2, L2 were 3.69, 1.47, and 0.74 mg/kg b.w., respectively) of halicin evaluated by H&E staining (40×, Scale bar: 100 μm).
